# Pyroptosis‐related molecular classification and immune microenvironment infiltration in breast cancer: A novel therapeutic target

**DOI:** 10.1111/jcmm.17247

**Published:** 2022-03-01

**Authors:** Jiayue Luo, Jianguo Lai

**Affiliations:** ^1^ Department of Breast Surgery Guangzhou Women and Children’s Medical Center Guangzhou Medical University Guangzhou Guangdong China; ^2^ Department of Breast Cancer Guangdong Provincial People’s Hospital Guangdong Academy of Medical Sciences Guangzhou Guangdong China

**Keywords:** breast cancer, immune microenvironment infiltration, pyroptosis, signature, therapeutic target

## Abstract

The underlying role of pyroptosis in breast cancer (BC) remains unknown. Herein, we investigated the correlations of 33 pyroptosis‐related genes (PRGs) with immune checkpoints and immune cell infiltrations in BC patients based on The Cancer Genome Atlas cohort (n = 996) and Gene Expression Omnibus cohort (n = 3,262). Enrichment analysis revealed that these PRGs mainly functioned in pyroptosis, inflammasomes and regulation of autophagy pathway. Four prognostic independent PRGs (CASP9, TIRAP, GSDMC and IL18) were identified. Then, cluster 1/2 was recognized using consensus clustering for these four PRGs. Patients from cluster 1 had a favourable prognosis and diverse immune cell infiltrations. A nomogram was developed based on age, TNM stage, tumour subtype and pyroptosis score. Patients with the high‐risk group exhibited worse 5‐year OS, and the result was consistent in the external cohort. Additionally, high‐risk group patients were associated with downregulated immune checkpoint expression. Further analysis suggested that the high‐risk group patients were associated with a higher IC50 of paclitaxel, doxorubicin, cisplatin, methotrexate and vinorelbine. In summarizing, the pyroptosis score‐based nomogram might serve as an independent prognostic predictor and could guide medication for chemotherapy. Additionally, it may bring novel insight into the regulation of tumour immune microenvironment in BC and help to achieve precision immunotherapy.

## 1 INTRODUCTION

Globally, breast cancer (BC) is one of the most common malignant tumours, which ranks first in females in terms of incidence.^1^, ^2^, ^3^ In the clinic, the treatment approach for nonmetastatic BC mainly depends on the clinical TNM stage and molecular subtypes. The standardized application of adjuvant systemic therapy can lower mortality from BC.^4^ However, almost 30% of nonmetastatic BC patients suffered from disease recurrence or metastasis after initial comprehensive treatments.^5^ Besides, numerous studies had revealed that BC was a heterogeneous group of cancer resulting in diverse phenotypes which had a variety of recurrence risks.^6^, ^7^, ^8^ Additionally, immunotherapy was applied in PD‐L1‐positive metastatic triple‐negative BC, but the detection and predictive value of PD‐L1 has limitations, and immunotherapy should be distinguished.^9^ Thus, it would be of great value to create clinically applicable predictive models to aid in identifying those patients with high risk and predicting the overall survival and immunotherapeutic response in advanced BC patients.

Pyroptosis, a form of programmed cell death apart from apoptosis and autophagy, is featured by cytoplasmic swelling, cells lysis and release of inflammatory factors, which triggered inflammation and immune response.^10^ The pyroptosis can be activated by the caspase‐1/3/4/5/11 inflammasome pathway and is induced by the gasdermin family.^11^, ^12^, ^13^, ^14^ Recent studies on pyroptosis had found that it may be closely related to malignant tumours and play a vital role in either antitumour effect or carcinogenesis, progression and drug resistance.^15^ For instance, studies had revealed that the overexpression of AIM2 inflammasome exhibited antitumour effects in hepatocellular cancer, and decreased expression or absence of AIM2 may cause colorectal carcinoma.^16^, ^17^ On the contrary, overexpression of GSDMB was reported to be associated with a worse prognosis and less sensitivity to HER2‐targeted therapy in BC.^18^ Besides, DFNA5/GSDME acted as an antioncogene in hepatocellular cancer, and loss of DFNA5/GSDME contributed to drug resistance in melanoma and lung cancer.^19^, ^20^, ^21^

Signatures based on pyroptosis showed substantial prognostic predictive values in ovarian cancer,^22^ glioblastoma,^23^ melanoma,^24^ and gastric cancer,^25^ but pyroptosis‐related signatures on BC are lacking. Besides, pyroptosis was identified in many types of immune cells, but the potential function of pyroptosis in the tumour immune microenvironment (TIME) remains undefined.^26^ A study demonstrated that pyroptosis‐produced cytokines were released into the TIME, exhibiting tumour‐promoting effects.^27^ However, pyroptosis‐induced inflammation in the TIME could enhance the sensitivity of immunotherapy through activating certain immune cells.^28^ Overall, existing studies had confirmed that pyroptosis had an essential role in both oncogenic and antioncogenic aspects, and we may utilize it as a prognosis predictor to help in making decisions in the treatment selected.

This study aimed to comprehensively evaluate the association of pyroptosis with the prognosis, TIME and chemotherapy responsiveness in BC patients. We constructed a PRGs‐based nomogram and estimated its efficacy in prognostic prediction, chemotherapeutic responsiveness and function in TIME regulation.

## 2 MATERIALS AND METHODS

### 2.1 Data extraction and identification of pyroptosis‐related genes

Gene expression files of breast normal and tumour tissues, clinicopathological characteristics and the corresponding follow‐up information of BC patients were extracted from The Cancer Genome Atlas (TCGA) data set (https://portal.gdc.cancer.gov/). Moreover, mutation information and copy number variation information for BC were also downloaded from the TCGA data set. The gene expression files and clinical information mentioned above of the external validation cohort were retrieved from the Gene Expression Omnibus (GEO) databank (https://www.ncbi.nlm.nih.gov/geo/). Analyses of these data were conducted by R (version 4.0.5) and R Bioconductor packages. Pyroptosis‐related genes (PRGs) were acquired from published literatures.^29^, ^30^, ^31^ Then, the Search Tool for the Retrieval of Interacting Genes (STRING), version 11.0 (https://string‐db.org/), was applied to construct a protein–protein interaction (PPI) network for the PRGs.

### 2.2 Functional enrichment analysis and mutation analysis of PRGs

We performed GO analysis in the heatmap and network of enriched terms on the Metascape website (http://metascape.org) to identify the biological process function of these selected PRGs. The ‘ggplot2’ package in R was applied to perform KEGG analysis. GSEA analysis was achieved in the Hallmark gene set with GSEA v4.1.0 software. Pathways were defined as enriched when *p* < 0.05.

### 2.3 Consensus clustering for the PRGs and immune cell infiltration (ICI) analysis

The unsupervised clustering analysis based on the expression levels of PRGs was conducted by the ‘Consensus Cluster Plus’ R package. The ‘CIBERSORT’ R package was utilized to clarify the distinct infiltration levels for immune cells and to quantify the percentage of 22 common immune cell types in the BC samples in different clusters and different risk groups. Spearman analysis was applied to investigate the correlation coefficient between any two types of immune cells.

### 2.4 Development and validation of pyroptosis risk score‐based nomogram

According to prior reviews, a total of 33 PRGs were identified.^15^, ^32^, ^33^ Next, univariate and multivariate Cox regression analyses (UMCRA) were implemented to search the correlation between the PRGs expression level and 5‐year OS of BC patients and to explore independent prognostic PRGs in the TCGA cohort. Specifically, univariate Cox regression analysis was utilized to screen out PRGs that were significantly related to the OS, and PRGs that meet the requirements of *p* < 0.05 were extracted for multivariate Cox regression analysis. Additionally, the pyroptosis risk score (PS) was established using the screening independent prognostic PRGs. The PS was calculated after centralization and standardization by applying the ‘scale’ function in R. Formulae are as follows: PS = expression level of PRGs × sum of coefficients. What’s more, Cox analysis was utilized to screen independent clinical variables for models. Finally, a novel PS‐based nomogram containing the PS, age, tumour subtype and TNM stage was developed. The AUC of the time‐dependent ROC curve and the calibration curve was conducted to weight the 5‐year OS prediction capability and the calibration capability of the PS‐based nomogram, separately. Data from the external cohort were analysed to further validate this model. Stratification analysis in subgroups was conducted to estimate the discriminative abilities for risk stratification of this model as well.

### 2.5 Assessment of the therapeutic response

To evaluate the sensitivity of drugs commonly treated for BC patients, we measured the IC50 of five chemotherapeutic agents and one small‐molecule inhibitor agent (paclitaxel, doxorubicin, cisplatin, lapatinib, methotrexate and vinorelbine) acquired from the GDSC website and the R package pRRophetic was applied to calculate the IC50 of these agents in the BC patients. Wilcoxon signed‐rank test was performed to compare the difference in the IC50 between the high‐ and low‐risk groups.

### 2.6 S**tatistical analysis**

Statistical analyses were carried out utilizing Stata/MP, version 14.0 (Stata Corp LP, College Station, TX), and R software (www.r‐project.org, version 3.6.1). The χ2 test and Fisher’s exact test were applied to compare the categorical variables in the primary and external validation cohorts. The Kruskal–Wallis test was utilized to calculate the differences of nomogram score across different subgroups which were stratified by following clinical characteristics such as tumour size, lymph node status, TNM stage and subtype. The Wilcoxon test was performed to compare the stromal score, estimated score, immune score and immune checkpoint expression levels between two risk groups. Pearson correlation tests were employed to finish the correlation analysis of immune infiltration levels. Kaplan–Meier survival analysis and a log‐rank test were utilized to draw the survival curve and to compare the difference between groups. The independent prognostic predictors were determined by UMCRA. Statistically significant was defined as the *p* value of analyses < 0.05.

## 3 RESULTS

### 3.1 Identification of PRGs between breast normal and tumour tissues

**FIGURE 1:**
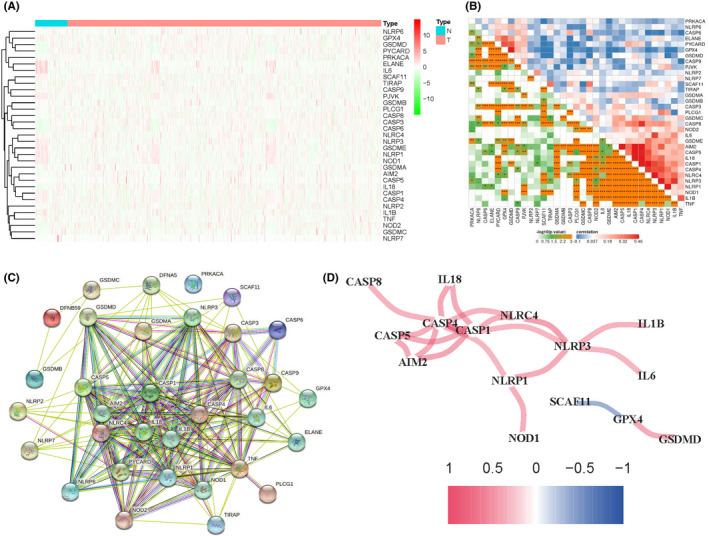
Correlations of pyroptosis‐related genes are differentially expressed between normal and tumour tissues. (A) The expression levels of the 33 PRGs between the BC tumour tissues (T) and the normal tissues (N) were shown as a heatmap. (B) The correlations (red represents positive correlation and blue represents negative correlation) of the 33 PRGs; P values were shown as: **p< 0.01; ***p < 0.001. (C) PPI network showed the interactions of the PRGs. (D) The correlation network of the PRGs

Firstly, we extracted PRG expression files of 111 normal and 1074 tumour tissues from the TCGA data set. Thirty‐three PRGs were selected based on the previous data.^29^, ^30^, ^31^ Thirty‐three PRGs were NLRP6, GPX4, GSDMD, PYCARD, PRKACA, ELANE, IL16, SCAF11, TIRAP, CASP9, PJVK, GSDMB, PLCG1, CASP8, CASP3, CASP6, NLRC4, NLRP3, GSDME, NLRP1, NOD1, GSDMA, AIM2, CASP5, IL18, CASP1, CASP4, NLRP2, IL1B, TNF, NOD2, GSDMC and NLRP7, respectively. The RNA expression levels of these 33 PRGs between normal and tumour tissue were illustrated as a heatmap (Figure 1A). Then, we used the spearman method to analyse the correlation coefficient between any two PRGs (Figure 1B). We identified that the expression levels of TNF, IL18, NLRP3, NLRP4, NOD1, NLRP1, CASP4, CASP1, CASP5 AIM2, CASP8 and GSDME were positively correlated with at least one of them. Next, we performed a protein–protein interaction (PPI) analysis to investigate the interactions of the 33 PRGs with the minimum required interaction score setting at 0.9 (Figure 1C). In addition, the correlation network (red: positive correlations; blue: negative correlations) of part of these PRGs that could link to each other was also presented (Figure 1D).

### 3.2 Functional enrichment analysis and mutation information of PRGs

To investigate the biological function and behaviour of the 33 PRGs, we performed GO analysis. The results revealed that these PRGs were chiefly involved in these terms: nucleotide‐binding oligomerization domain (NOD) pathway, pyroptosis, inflammasomes, protein processing, apoptotic signalling pathway, necrotic cell death, inflammatory response to an antigenic stimulus, regulation of autophagy and negative regulation of NF‐kappa B transcription factor activity (Supplementary Figure 1A‐B). Moreover, some genetic alterations were detected in these 33 PRGs. Of these, GSDMA, GSDMB, GSDMC, GSDMD, AIM2 and NLRP3 showed a high frequency of amplification (Supplementary Figure 1).

### 3.3 Construction of prognostic‐related PRGs risk score in BC patients

UMCRA were utilized to screen independent prognostic‐related PRGs at the base of 33 PRGs. After the analysis, four PRGs were recognized to be significantly correlated with the survival of BC patients, including CASP9, TIRAP, GSDMC and IL18. Then, four PRGs were applied to calculate the pyroptosis score (PS). A PS formula was constructed based on the expression levels of PRGs and their estimated regression coefficients, where PS = GSDMC*0.035‐CASP9*0.262‐IL18*0.084+TIRAP*0.168.

### 3.4 Consensus clustering analysis based on the PRGs

A consensus clustering analysis with 996 BC patients in the TCGA cohort was conducted to explore the correlations between the four PRGs expression levels and BC subtypes. The most appropriate clustering number of k between 2 and 9 was assessed, and k = 2 was selected as the excellent clustering number based on the cumulative distribution function (CDF) value and delta area (Supplementary Figure 2A‐C). Specifically, 138 cases were grouped to cluster 1 and 858 cases were grouped to cluster 2. The 5‐year overall survival rate of cluster 2 was significantly poorer than that of cluster 1 (Supplementary Figure 2D).

### 3.5 Differences in clinical features and ICI between the two clusters

The clinicopathological features consisted of the tumour size (T1‐T4), lymph node status (N0‐N3), TNM stages (I–IV) and survival status (alive or dead) were presented in a heatmap (Supplementary Figure 2E). Then, we analysed the distribution differences of ICI between two clusters and found that M1 macrophages, activated CD4 memory T cells, CD8 T cells and follicular helper T cells were enriched in cluster 1 while M0 macrophages, resting T4 memory cells, resting mast cells and M2 macrophages were abundant in cluster 2 (Supplementary Figure 2F). The result indicated that activated innate ICI was distributed much more in cluster 1, thus conferring a better survival.

### 3.6 Establishment and validation of the PS‐based nomogram

**FIGURE 2:**
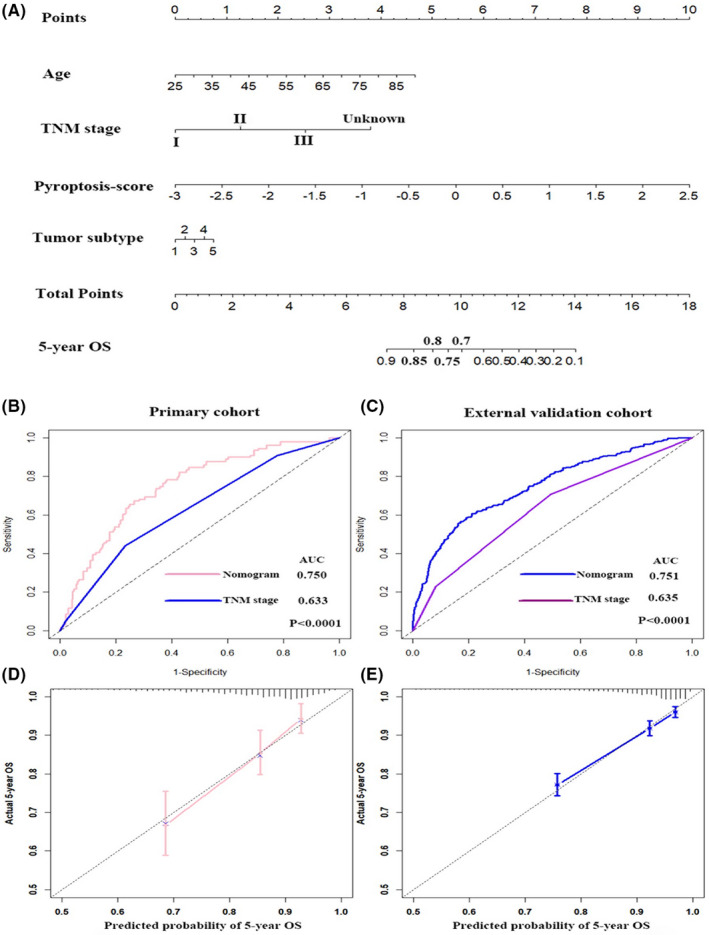
Nomogram for predicting the 5‐year OS of patients in the primary cohort (A). ROC curve and calibration plot for validating the accuracy of the nomogram in the primary cohort (B, D) and the external validation cohort (C, E)

A total of 996 BC patients were extracted from the TCGA cohort as the primary cohort, and 3,262 BC patients were acquired from the GEO cohort (GSE96058) as the external validation cohort. No significant differences were detectable in the baseline characteristics between these two cohorts (all *p* > 0.05; Table 1). Next, UMCRA of clinical variables and PS were conducted and the results are summarized in Table 2. It demonstrated that age, TNM stage, tumour subtype and PS were independent prognostic predictors. Therefore, to further create a clinical tool as applied to predict the 5‐year OS, we constructed a PRGs‐related nomogram based on the patient’s age, TNM stage, tumour subtype and pyroptosis score in the primary cohort and it was validated in the external validation cohort (Figure 2A).

**TABLE 1:** Baseline characteristics of the included patients

Variables	Primary cohort	External validation cohort
	No. (%)	No. (%)
No. of patients	996(100%)	3,262(100%)
Age (years)	58(48,67)	64(53,71)
T stage		
T1	271(27.2)	2,124(65.1)
T2	565(56.7)	1,060(32.5)
T3	124(12.5)	78(2.4)
T4	36(3.6)	0(0)
N stage		
N0	463(46.5)	2,079(63.7)
N1	341(34.2)	887(27.2)
N2	105(10.6)	268(8.2)
N3	70(7.0)	28(0.9)
Unknown	17(1.7)	0(0)
TNM stage		
I	167(16.8)	1,534(47.0)
II	575(57.7)	1,409(43.2)
III	237(23.8)	319(9.8)
Unknown	17(1.7)	0(0)
ER status		
Negative	207(20.8)	237(7.3)
Positive	752(75.5)	2,830(86.7)
Unknown	37(3.7)	195(6.0)
PR status		
Negative	298(29.9)	385(11.8)
Positive	660(66.3)	2,554(78.3)
Unknown	38(3.8)	323(9.9)
HER2 status		
Negative	697(70.0)	2,746(84.2)
Positive	175(17.6)	404(12.4)
Unknown	124(12.4)	112(3.4)
Tumour subtype		
HR+/HER2‐	559(56.1)	2,354(72.2)
HR+/HER2+	139(14.0)	277(8.5)
HR‐/HER2+	36(3.6)	53(1.6)
TNBC	137(13.8)	146(4.5)
Unknown	125(12.5)	432(13.2)

Abbreviations

ER, oestrogen receptor; HER2, human epidermal growth factor receptor 2; PR, progesteron receptor; TNBC, triple‐negative breast cancer; TNM, tumour, node, metastasis.

**TABLE 2:** Univariate and multivariate analyses in the primary cohort

Variables	Univariate analysis		Multivariate analysis	
	HR (95%CI)	*P* value	HR (95%CI)	*P* value
Age	1.032(1.018–1.046)	**<0.001**	1.035(1.021– 1.049)	**<0.001**
T stage				
T1	Referent			
T2	1.194(0.789–1.808)	0.401		
T3	1.231(0.699–2.168)	0.472		
T4	2.555(1.348–4.842)	**0.004**		
N stage				
N0	Referent			
N1	1.826(1.217–2.742)	**0.004**		
N2	2.683(1.553–4.634)	**<0.001**		
N3	4.152(2.207–7.810)	**<0.001**		
Unknown	6.515(3.155–13.456)	**<0.001**		
TNM stage				
I	Referent		Referent	
II	1.545(0.880–2.713)	0.130	1.435(0.812–2.538)	0.214
III	3.104(1.737–5.546)	**<0.001**	2.980(1.648– 5.389)	**<0.001**
Unknown	7.300(3.187–16.726)	**<0.001**	5.333(2.235–12.727)	**<0.001**
Tumour subtype				
HR+/HER2‐	Referent		Referent	
HR+/HER2+	1.460(0.830–2.567)	0.189	1.360(0.765–2.417)	0.294
HR‐/HER2+	2.224(0.951–5.198)	0.065	1.269(0.526–3.062)	0.597
TNBC	1.587(0.953–2.643)	0.076	1.872(1.119–3.130)	**0.017**
Unknown	1.682(1.080–2.618)	**0.021**	1.320(0.849–2.054)	0.218
Pyroptosis score	2.724(1.908–3.888)	**<0.001**	2.330(1.655–3.278)	**<0.001**

Abbreviations

ER, oestrogen receptor; HER2, human epidermal growth factor receptor 2; PR, progesteron receptor; TNBC, triple‐negative breast cancer; TNM, tumour, node, metastasis.

Bold values are statistically significant differences.

The time‐dependent receiver operating characteristic (ROC) curves were utilized to evaluate the 5‐year OS predictive ability of our nomogram and TNM stage in the primary cohort and external validation cohort (Figure 2B, C). The results revealed that the area under the ROC curve (AUC) was 0.750 for nomogram, 0.633 for TNM stage in the primary cohort and 0.751 for nomogram and 0.635 for TNM stage in the external validation cohort (all *p *< 0.001), indicating that the nomogram had superior accuracy comparing with TNM stage in both cohorts. What’s more, the calibration plot found excellent concordance between the prediction model value by nomogram and the actual observation value of the 5‐year OS in BC in both cohorts (Figure 2D, E).

### 3.7 Clinical evaluation by PS‐based nomogram score

**FIGURE 3:**
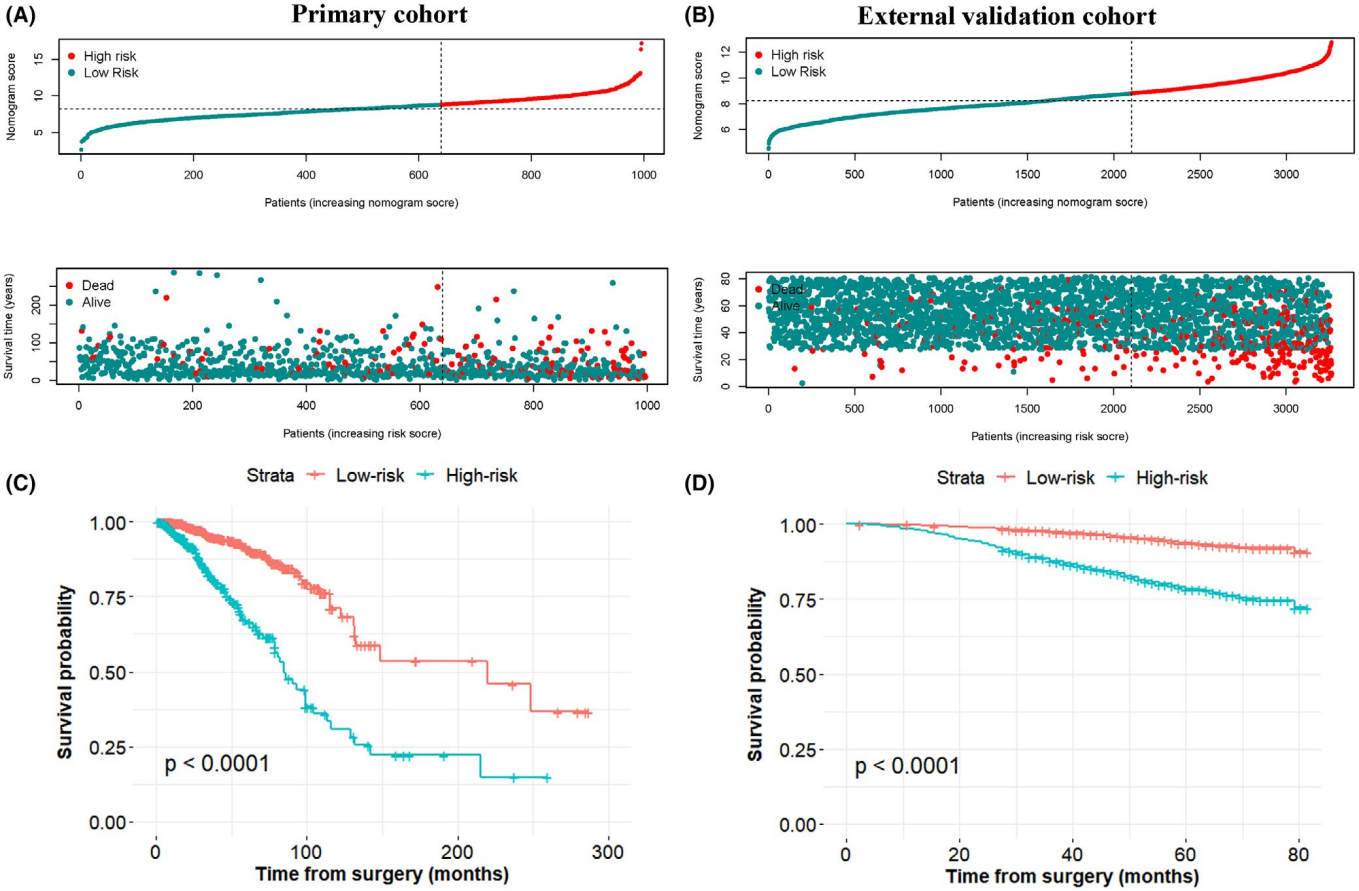
Distribution of patients in the primary cohort (A) and external validation cohort (B) based on the median risk score and survival status of each patient. Kaplan–Meier curves showed the OS between low‐ and high‐risk groups in the primary cohort (C) and external validation cohort (D)

By using the optimal nomogram score of each cohort as cut‐off values, we separated the BC patients into a low‐risk group (N = 640) and a high‐risk group (N = 356). The distribution of risk score and the survival status of each case are shown in Figure 3A (primary cohort) and Figure 3B (external validation cohort). As the nomogram score raised, more deaths happened and the survival time of patients declined. Kaplan–Meier analysis suggested that 5‐year OS in the low‐risk group patients was more favourable than that in high‐risk group patients in both the primary cohort and the external validation cohort (*p* < 0.0001) (Figure 3C, D).

### 3.8 The nomogram retains its ability to predict OS in stratification analysis

**FIGURE 4:**
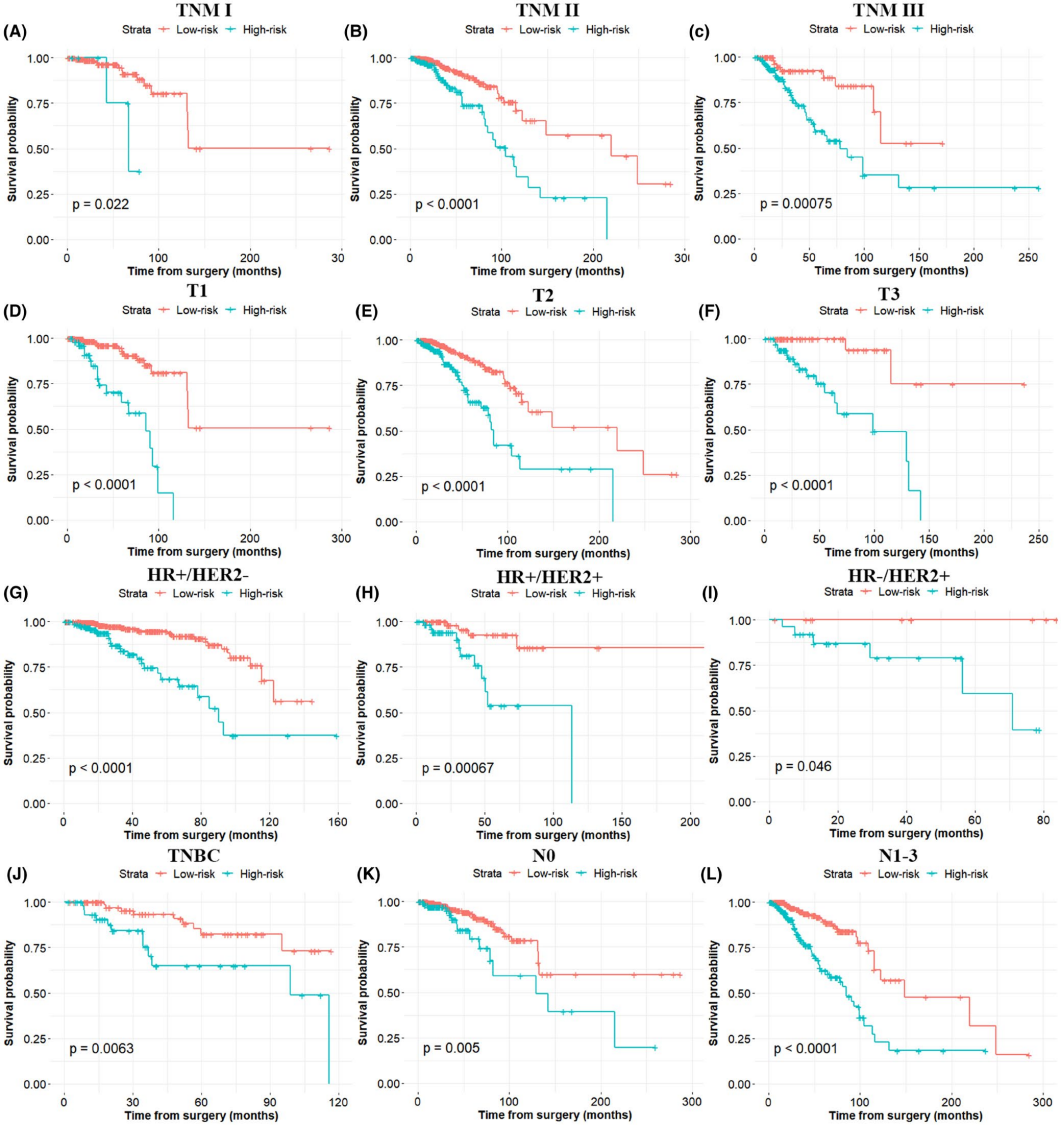
Nomogram retained its prognostic capacity in various subgroups of BC patients (including patients with TNM stage I, TNM stage II, TNM stage III, T1, T2, T3, N0, N1‐3, luminal subtype, triple‐positive subtype, HER2‐positive (hormonal receptor‐negative) subtype and triple‐negative subtype). All *p* < 0.05

To better understand the relationship between clinical features and the nomogram risk model in the primary cohort, a stratification analysis was conducted to determine whether the nomogram retained its prognostic prediction ability in diverse subgroups. As shown in Figure 4, in contrast with the low‐risk group patients, high‐risk group patients had worse OS in TNM stage (I, II and III), tumour size (T1, T2 and T3), N0 and N1‐3 subgroups, respectively. Likewise, the nomogram also retained its prognostic prediction capacity for different molecular subtyping of BC, such as luminal subtype, triple‐positive subtype, HER2‐positive (hormonal receptor‐negative) subtype and triple‐negative subtype. As expected, in these molecular subtypes, all patients in the low‐risk group had a survival advantage, compared to that in the high‐risk group. These data suggested that the nomogram had the potential to predict OS for BC patients.

### 3.9 Associations between clinical features and the levels of nomogram score

**FIGURE 5:**
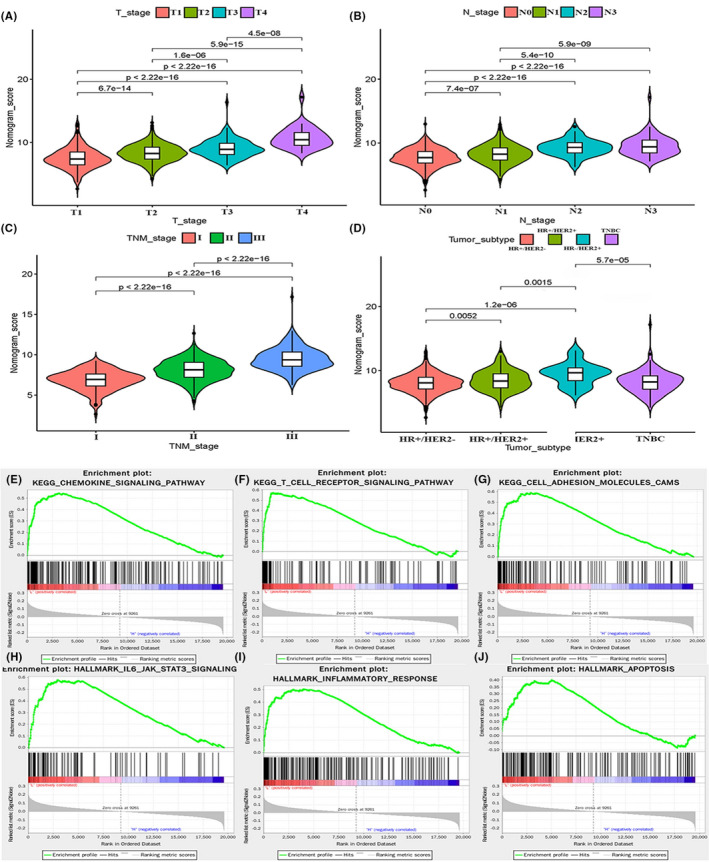
Patients with different clinical features (including T stage (A), N stage (B), TNM stage (C) and tumour subtype (D)) had significantly different levels of nomogram scores. GSEA showed that the chemokine signalling pathway (E), T cell receptor signalling pathway (F), cell adhesion molecules CAMS (G), IL6/JAK/STAT3 signalling (H), inflammatory response (I) and apoptosis (J) are differentially enriched in the low‐risk group

A series of Wilcoxon rank tests were performed to explore the correlation between the clinical characteristics and the different levels of nomogram score. Results revealed that with an increase in the levels of nomogram score, patients experienced significantly worse clinical stages including T stage, N stage and TNM stage (Figure 5A‐C). Specifically, T4, N3 and TNM‐III stages had the highest nomogram scores. For molecular subtypes, the HR‐/HER+subtype had higher nomogram scores than all the others, while HR+/HER2‐ subtype had the lowest scores (Figure 5D).

### 3.10 Gene set enrichment analysis of PRGs in different risk groups

To further investigate the potential molecular mechanisms leading to the differences between low‐risk and high‐risk groups, the gene set enrichment analysis (GSEA) was utilized. The results revealed that several malignant hallmarks were enriched in the low‐risk group, including chemokine signalling pathway, IL6/JAK/STAT3 signalling, T cell receptor signalling pathway, inflammatory response, cell adhesion molecules CAMS and apoptosis (Figure 5E‐J). Hence, the inflammatory response and apoptosis might be associated with pyroptosis, and IL6/JAK/STAT3 pathway may be implicated in the different prognoses of these two risk groups.

### 3.11 The landscape of immune cell infiltration in risk subtypes

**FIGURE 6:**
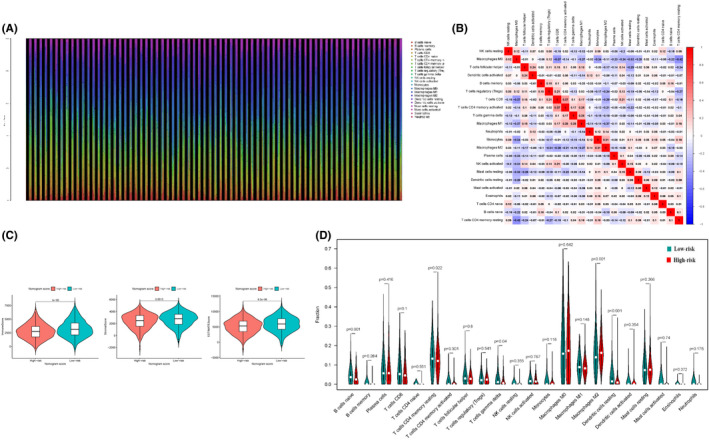
Landscape of ICI in risk subtypes. (A) The relative proportion of ICI in BC subtypes. (B) Correlation coefficients are represented in the form of a heatmap using coloured scales ranging from blue (negative correlation) to red (positive correlation). (C) Comparison of the two risk subtypes in three immune‐related indicators such as immune score, estimate score and stromal score. (D) Violin plots of the proportions of 22 immune infiltrating cells in tumour tissues with low‐risk (green) or high‐risk (red) groups

CIBERSORT algorithm, including the gene expression levels, was utilized to investigate the differences of immune cell infiltration (ICI) between the two risk groups of BC patients. We found that the significant differences of the 22 immune cell proportion existed between the two groups (Figure 6A). Then, to investigate the correlation coefficient values between any two types of immune cells, we used the spearman method and found that activated CD4 memory cells were positively related to CD8 T cells (*p* < 0.001, r = 0.37), while macrophage M0 was negatively correlated with resting CD4 memory T cells (*p* < 0.001, r = −0.42) (Figure 6B). In the next step, three indicators related to BC immunogenicity including immune score, estimate score and stromal score were computed by the expression data (ESTIMATE) algorithm in both groups. All three indicators were evidently higher in the low‐risk group (Figure 6C), indicating that low‐risk group patients may have a better response to immune therapy. Additionally, we investigated the distinct distributions of ICI between the two risk groups. As displayed in Figure 6D, the low‐risk group had an abundance of naive B cells, resting CD4 memory T cells and resting dendritic cells. In contrast, the high‐risk group had a notably higher proportion of macrophage M2. These results indicated that the heterogeneity of ICI between high‐ and low‐risk groups may encompass targets for immunotherapy.

### 3.12 The association between immune checkpoint genes and risk groups

**FIGURE 7:**
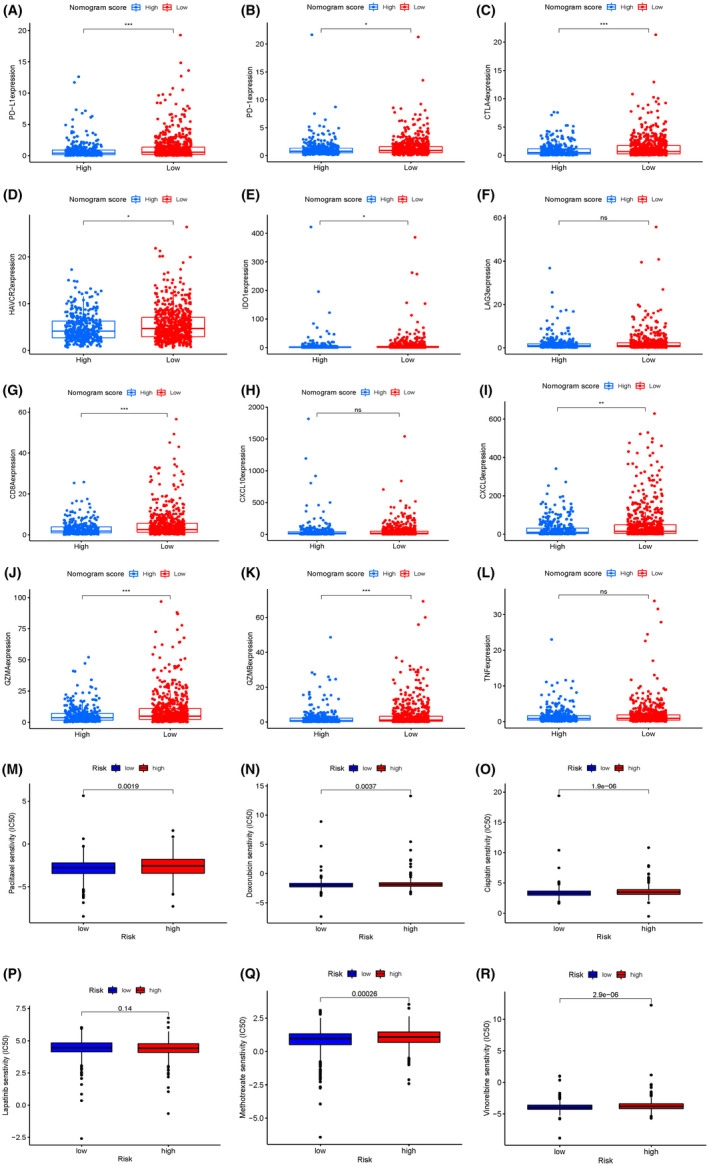
Differential expression of immune checkpoint genes (including PD‐L1, PD‐1, CTLA4, HAVCR2, IDO1, LAG3, CD8, CXCL10, CXCL9, GZMA, GZMB and TNF) in the low‐risk group and high‐risk group of the primary cohort (A‐L). The estimated IC50 of paclitaxel (M), doxorubicin (N), cisplatin (O), lapatinib (P), methotrexate (Q) and vinorelbine (R) between the two risk groups in the primary cohorts

To assess whether different risk groups had distinct sensitivity to immunotherapy, we also explored the expression levels alteration of immune checkpoints. Specifically, the differential expression levels of immune checkpoints were compared between different risk groups in the primary cohort (Figure 7A‐L). The analysis displayed that the low‐risk group had higher expression levels of PD‐L1, PD‐1, CTLA4, HAVCR2, IDO1, CD8, CXCL9, GZMA and GZMB, thus revealing a better response to immunotherapy. However, no significant difference was found in the expression levels of LAG3, CXCL10 and TNF.

### 3.13 Assessment of the sensitivity of therapeutic agents

Most early BC patients need to accept chemotherapy to reduce the risks of recurrence and metastasis. However, not all of these patients were sensitive to these drugs. Thus, we selected five chemotherapeutic agents and one small‐molecule inhibitor agent (paclitaxel, doxorubicin, cisplatin, lapatinib, methotrexate and vinorelbine) that might benefit patients in the high‐risk group. Then, the estimated half inhibitory centration (IC50) value of these drugs was compared and the result revealed that patients in the high‐risk group were associated with a higher IC50 of paclitaxel, doxorubicin, cisplatin, methotrexate and vinorelbine, whereas it was associated with lower IC50 for lapatinib (Figure 7M‐R). The results implied that this model might serve as a predictor for chemotherapy responsiveness.

## 4 DISCUSSION

Pyroptosis has a two‐way effect in cancers. Stimulation by pyroptosis releasing inflammatory factors can transform normal cells into tumour cells.^33^ In contrast, pyroptosis can facilitate cancer cell death, thus play an antitumour role.^34^ In BC, different expression levels of pyroptosis‐related cytokines, including inflammasome, gastermin family and caspase may lead to exactly opposite effects. However, the expression levels of several gastermins did not appear to act as reliable prognostic factors. The role of PRG signature in BC has not yet been elucidated. Therefore, a prognostic signature by combining independent prognostic PRGs in BC needed to explore.

In our study, the expression patterns, biological functions and genetic alterations of 33 PRGs were illustrated. As expected, functional analyses showed that these PRGs were chiefly enriched in cell death‐related pathways such as pyroptosis, necrotic cell death, apoptotic signalling pathway and regulation of autophagy. Additionally, they were also enriched in the cancer‐related NF‐κB pathway. However, only four (CASP9, TIRAP, GSDMC and IL18) independent prognostic predictors were finally confirmed for further analysis. Among these four PRGs, CASP9 might act as anti‐oncogenes while TIRAP, IL‐18 and GSDMC might be oncogenes. Upregulation of CASP9 by miR‐182‐5p inhibition can induce apoptosis and antiproliferative effect in breast cancer.^35^ Similarly, upregulation of CASP9 through NF‐κB contributed to apoptosis activation in cancer cells, thus exerting the antitumour effect.^36^ On the contrary, downregulation of CASP9 by microRNA‐224^37^ or inactivation of CASP9 by lncRNA POU3F3^38^ promoted proliferation and progression in TNBC. TIRAP was reported to be an upstream regulator of the NF‐κB pathway and its expression enhanced cell proliferation in lymphoma.^39^ Another study revealed that downregulation of TIRAP contributed to the anti‐proliferation effect in non‐small‐cell lung cancer cells.^40^ Studies about GSDMC suggested that it functioned as an oncogene, facilitating cell proliferation in colorectal cancer^41^ and serving as an independent prognostic predictor in lung adenocarcinoma.^42^ Besides, GSDMC was found to be highly expressed in BC and correlated with poorer survival.^43^ A previous study reported that tumour‐derived IL18 could induce PD‐1 expression on NK cells, which resulted in a poor prognosis in patients with TNBC.^44^ Under the stimulation of leptin, IL18 expression increased and promoted BC cell migration and invasion.^45^ Another study reported a similar conclusion that high serum levels of IL18 resulted in worse outcomes in patients with BC.^46^ These results showed that different expression levels of PRGs might act as a protective role or a disrupted role in cancer.

After consensus clustering for the four PRGs, two subtypes of BC, namely cluster 1 and cluster 2, were identified. The cluster 1/2 had distinct prognoses, clinical features and ICIs of BC. We also calculated the pyroptosis score from the four PRGs and subsequently constructed a nomogram encompassed PS, age, TNM stage and tumour subtypes. This nomogram harboured the ability to separate the OS of BC patients into low‐ and high‐risk groups in both TCGA and GEO cohorts. As was expected, high‐risk group patients had worse 5‐year OS in all BC patients and diverse subgroups stratified by TNM stage (I, II and III), tumour size (T1, T2 and T3), N0 and N1‐3, compared to that of low‐risk group patients. Moreover, patients in the high‐risk group revealed a larger tumour size, higher histological grade, more metastatic lymph nodes, worse TNM stages and worse molecular subtypes, compared to that of the low‐risk group. Additionally, the GSEA results implicated that cell death‐related hallmarks (the inflammatory response and apoptosis pathways) and malignant hallmarks (IL6/JAK/STAT3 signalling pathway) were significantly enriched in low‐risk groups. Previous research demonstrated that gasterminB could interact with STAT3 and activate STAT3 signalling, which promotes tumour cell growth in bladder cancer.^47^ Hence, pyroptosis‐related genes and the IL6/JAK/STAT3 signalling pathway may be jointly involved in the modulation of oncogenesis in BC.

The tumour immune microenvironment (TIME) is reported to play a significant role in BC. The heterogeneity of TIME may affect the therapeutic response, resulting in different clinical outcomes.^48^ A previous study reported that distinct tumour microenvironment features were associated with distinct OS in patients with TNBC.^49^ In our study, the different risk groups had different ICI and immune checkpoint expression levels. The low‐risk group had an abundance infiltration level of resting CD4 memory T cell, naive B cell and resting dendritic cells. Previous research reported that these immune cells are protective against tumour growth.^50^ Recently, a study reported that the transcription of the GSDMC gene can be enhanced by PD‐L1, which subsequently switches TNF α‐induced apoptosis to pyroptosis.^43^ IL1 β was reported to be an inflammasome that can drive CD8+ T cell responses, increase the numbers of CD4+ T cells and inhibit the immunosuppressive T regulatory cells to differentiate.^51^ Another research found that IL18 acts as an important role in Th‐1 polarization and natural killer (NK) cell recruitment and activation.^52^ Besides, IL‐18 also participated in the activation and recruitment of dendritic cells and macrophages.^53^ These findings suggested that PRGs are involved in TIME regulation by affecting the infiltration level of immune cells. Our study also revealed that the low‐risk group patients had a higher immune score, estimate score and stromal score. Besides, higher expression levels of PD‐L1, PD‐1, CTLA4, HAVCR2, IDO1, CD8, CXCL9, GZMA and GZMB also existed in the low‐risk group. Bringing up together, low‐risk group BC patients may have a better treatment response to immune therapy.

To test the responsiveness to commonly recommended chemotherapeutic agents, five agents including paclitaxel, doxorubicin, cisplatin, lapatinib, methotrexate and vinorelbine were assessed. We found that the estimated IC50 values of paclitaxel, cisplatin, doxorubicin, methotrexate and vinorelbine were distinctly higher in the high‐risk group. As we know, paclitaxel and doxorubicin are standard agents used in the treatment of early BC, while vinorelbine is commonly used in metastatic BC.^4^ For high‐risk group patients, paclitaxel, doxorubicin and vinorelbine may not be enough to reduce the recurrence or progression risk and additional enhanced treatment might need. This result brought the information that this model might serve as a predictor for chemotherapy responsiveness.

Our study still had certain limitations. Besides, data about how do pyroptosis affects TIME in BC are limited and deserve further research to find out the regulatory mechanism. In addition, in vitro and in vivo experiments are warranted to confirm the findings in our study.

## 5 CONCLUSION

In summary, our study comprehensively evaluated the prognostic value, functions in TIME regulation, and potential therapeutic efficacy of chemotherapy and immunotherapy in BC. The results of this study suggested that a novel signature established on the foundation of PRGs could predict clinical outcomes in BC patients, guide medication of chemotherapy and might help in screening those who could benefit from immunotherapy.

## CONFLICT OF INTEREST

The authors declare that they have no competing interests.

## AUTHOR CONTRIBUTIONS

**Jiayue Luo:** Formal analysis (equal); Funding acquisition (equal); Investigation (equal); Supervision (equal); Writing – original draft (equal). **Jianguo Lai:** Conceptualization (equal); Data curation (equal); Formal analysis (equal); Funding acquisition (equal); Investigation (equal); Supervision (equal); Writing – review & editing (equal).

## Supporting information

Supplementary Material

## Data Availability

The data sets for this study can be found in The Cancer Genome Atlas (TCGA) data set (https://portal.gdc.cancer.gov/) and the Gene Expression Omnibus (GEO) databank (https://www.ncbi.nlm.nih.gov/geo/).

## References

[1] Siegel RL , Miller KD , Fuchs HE , Jemal A . Cancer statistics, 2021. CA Cancer J Clin. 2021;71:7‐33. 33433946 10.3322/caac.21654

[2] Lai J , Chen B , Zhang G , Li X , Mok H , Liao N . Molecular characterization of breast cancer: a potential novel immune‐related lncRNAs signature. J Transl Med. 2020;18(1):416. 33160384 10.1186/s12967-020-02578-4 PMC7648293

[3] Li W , Xu M , Li Y , et al. Comprehensive analysis of the association between tumor glycolysis and immune/inflammation function in breast cancer. J Transl Med. 2020;18:92. 32070368 10.1186/s12967-020-02267-2 PMC7029444

[4] Waks AG , Winer EP . Breast cancer treatment: a review. JAMA. 2019;321:288‐300. 30667505 10.1001/jama.2018.19323

[5] Saphner T , Tormey DC , Gray R . Annual hazard rates of recurrence for breast cancer after primary therapy. J Clin Oncol. 1996;14:2738‐2746. 8874335 10.1200/JCO.1996.14.10.2738

[6] Zhao N , Rosen J . Breast cancer heterogeneity through the lens of single‐cell analysis and spatial pathologies. Semin Cancer Biol. 2021. doi:10.1016/j.semcancer.2021.07.010 PMC8761219 34274486

[7] Bareche Y , Venet D , Ignatiadis M , et al. Unravelling triple‐negative breast cancer molecular heterogeneity using an integrative multiomic analysis. Ann Oncol off J Eur Soc Med Oncol. 2018;29:895‐902. 10.1093/annonc/mdy024 PMC5913636 29365031

[8] Zardavas D , Irrthum A , Swanton C , Piccart M . Clinical management of breast cancer heterogeneity. Nat Rev Clin Oncol. 2015;12:381‐394. 25895611 10.1038/nrclinonc.2015.73

[9] Emens LA , Adams S , Barrios CH , et al. First‐line atezolizumab plus nab‐paclitaxel for unresectable, locally advanced, or metastatic triple‐negative breast cancer: IMpassion130 final overall survival analysis. Ann Oncol. 2021;32:983‐993. 34272041 10.1016/j.annonc.2021.05.355

[10] Fink SL , Cookson BT . Apoptosis, pyroptosis, and necrosis: mechanistic description of dead and dying eukaryotic cells. Infect Immun. 2005;73:1907‐1916. 15784530 10.1128/IAI.73.4.1907-1916.2005 PMC1087413

[11] Shi J , Zhao Y , Wang K , et al. Cleavage of GSDMD by inflammatory caspases determines pyroptotic cell death. Nature. 2015;526:660‐665. 26375003 10.1038/nature15514

[12] Kayagaki N , Stowe IB , Lee BL , et al. Caspase‐11 cleaves gasdermin D for non‐canonical inflammasome signalling. Nature. 2015;526:666‐671. 26375259 10.1038/nature15541

[13] Agard NJ , Maltby D , Wells JA . Inflammatory stimuli regulate caspase substrate profiles. Mol Cell Proteomics. 2010;9:880‐893. 20173201 10.1074/mcp.M900528-MCP200 PMC2871421

[14] Wang Y , Gao W , Shi X , et al. Chemotherapy drugs induce pyroptosis through caspase‐3 cleavage of a gasdermin. Nature. 2017;547:99‐103. 28459430 10.1038/nature22393

[15] Xia X , Wang X , Cheng Z , et al. The role of pyroptosis in cancer: pro‐cancer or pro‐"host"? Cell Death Dis. 2019;10:650. 31501419 10.1038/s41419-019-1883-8 PMC6733901

[16] Ma X , Guo P , Qiu Y , et al. Loss of AIM2 expression promotes hepatocarcinoma progression through activation of mTOR‐S6K1 pathway. Oncotarget. 2016;7:36185‐36197. 27167192 10.18632/oncotarget.9154 PMC5094992

[17] Dihlmann S , Tao S , Echterdiek F , et al. Lack of Absent in Melanoma 2 (AIM2) expression in tumor cells is closely associated with poor survival in colorectal cancer patients. Int J Cancer. 2014;135:2387‐2396. 24729378 10.1002/ijc.28891

[18] Hergueta‐Redondo M , Sarrió D , Molina‐Crespo Á , et al. Gasdermin‐B promotes invasion and metastasis in breast cancer cells. PLoS One. 2014;9:e90099. 24675552 10.1371/journal.pone.0090099 PMC3967990

[19] Wang CJ , Tang L , Shen DW , et al. The expression and regulation of DFNA5 in human hepatocellular carcinoma DFNA5 in hepatocellular carcinoma. Mol Biol Rep. 2013;40:6525‐6531. 24154762 10.1007/s11033-013-2581-8

[20] Lage H , Helmbach H , Grottke C , Dietel M , Schadendorf D . DFNA5 (ICERE‐1) contributes to acquired etoposide resistance in melanoma cells. FEBS Lett. 2001;494:54‐59. 11297734 10.1016/s0014-5793(01)02304-3

[21] Lu H , Zhang S , Wu J , et al. Molecular targeted therapies elicit concurrent apoptotic and GSDME‐dependent pyroptotic tumor cell death. Clin Cancer Res. 2018;24:6066‐6077. 30061362 10.1158/1078-0432.CCR-18-1478

[22] Ye Y , Dai Q , Qi H . A novel defined pyroptosis‐related gene signature for predicting the prognosis of ovarian cancer. Cell Death Discov. 2021;7:71. 33828074 10.1038/s41420-021-00451-x PMC8026591

[23] Li XY , Zhang LY , Li XY , Yang XT , Su LX . A pyroptosis‐related gene signature for predicting survival in glioblastoma. Front Oncol. 2021;11:697198. 34485134 10.3389/fonc.2021.697198 PMC8416108

[24] Ju A , Tang J , Chen S , Fu Y , Luo Y . Pyroptosis‐related gene signatures can robustly diagnose skin cutaneous melanoma and predict the prognosis. Front Oncol. 2021;11:709077. 34327145 10.3389/fonc.2021.709077 PMC8313829

[25] Shao W , Yang Z , Fu Y , et al. The pyroptosis‐related signature predicts prognosis and indicates immune microenvironment infiltration in gastric cancer. Front Cell Developm Biol. 2021;9:676485. 10.3389/fcell.2021.676485 PMC8226259 34179006

[26] Zychlinsky A , Prevost MC , Sansonetti PJ . Shigella flexneri induces apoptosis in infected macrophages. Nature. 1992;358:167‐169. 1614548 10.1038/358167a0

[27] Binnewies M , Roberts EW , Kersten K , et al. Understanding the tumor immune microenvironment (TIME) for effective therapy. Nat Med. 2018;24:541‐550. 29686425 10.1038/s41591-018-0014-x PMC5998822

[28] Minton K . Pyroptosis heats tumour immunity. Nat Rev Immunol. 2020;20:274‐275. 10.1038/s41577-020-0297-2 32203327

[29] Ye Y , Dai Q , Qi H . A novel defined pyroptosis‐related gene signature for predicting the prognosis of ovarian cancer. Cell Death Discov. 2021;7(1):71. 33828074 10.1038/s41420-021-00451-x PMC8026591

[30] Lin W , Chen Y , Wu B , Chen Y , Li Z . Identification of the pyroptosisrelated prognostic gene signature and the associated regulation axis in lung adenocarcinoma. Cell Death Discov. 2021;7:161. 34226539 10.1038/s41420-021-00557-2 PMC8257680

[31] Meng J , Huang X , Qiu Y , et al. Pyroptosis‐related gene mediated modification patterns and immune cell infiltration landscapes in cutaneous melanoma to aid immunotherapy. Aging. 2021;13(21):24379‐24401. 34753832 10.18632/aging.203687 PMC8610130

[32] Latz E , Xiao TS , Stutz A . Activation and regulation of the inflammasomes. Nat Rev Immunol. 2013;13:397‐411. 23702978 10.1038/nri3452 PMC3807999

[33] Karki R , Kanneganti TD . Diverging inflammasome signals in tumorigenesis and potential targeting. Nat Rev Cancer. 2019;19:197‐214. 30842595 10.1038/s41568-019-0123-y PMC6953422

[34] Ruan J , Wang S , Wang J . Mechanism and regulation of pyroptosis‐mediated in cancer cell death. Chem‐Biol Interact. 2020;323:109052. 32169591 10.1016/j.cbi.2020.109052

[35] Sharifi M , Moridnia A . Apoptosis‐inducing and antiproliferative effect by inhibition of miR‐182‐5p through the regulation of CASP9 expression in human breast cancer. Cancer Gene Ther. 2017;24:75‐82. 28084318 10.1038/cgt.2016.79

[36] Zhou F , Li Y , Huang Y , et al. Upregulation of CASP9 through NF‐κB and its target MiR‐1276 contributed to TNFα‐promoted apoptosis of cancer cells induced by doxorubicin. Int J Mol Sci. 2020;21:2290. 10.3390/ijms21072290 PMC7177739 32225068

[37] Zhang L , Zhang X , Wang X , He M , Qiao S . MicroRNA‐224 promotes tumorigenesis through downregulation of caspase‐9 in triple‐negative breast cancer. Dis Markers. 2019;2019:7378967. 30886656 10.1155/2019/7378967 PMC6388334

[38] Yang J , Meng X , Yu Y , Pan L , Zheng Q , Lin W . LncRNA POU3F3 promotes proliferation and inhibits apoptosis of cancer cells in triple‐negative breast cancer by inactivating caspase 9. Biosci Biotechnol Biochem. 2019;83:1117‐1123. 30843771 10.1080/09168451.2019.1588097

[39] Burkhard R , Keller I , Arambasic M , et al. R81C is a novel lymphoma risk variant which enhances cell proliferation via NF‐κB mediated signaling in B‐cells. Haematologica. 2019;104:766‐777. 30381301 10.3324/haematol.2018.201590 PMC6442981

[40] Hao S , Li S , Wang J , et al. Phycocyanin exerts anti‐proliferative effects through down‐regulating TIRAP/NF‐κB activity in human non‐small cell lung cancer cells. Cells. 2019;8:588. 10.3390/cells8060588 PMC6627414 31207932

[41] Miguchi M , Hinoi T , Shimomura M , et al. Gasdermin C is upregulated by inactivation of transforming growth factor β receptor type II in the presence of mutated apc, promoting colorectal cancer proliferation. PLoS One. 2016;11:e0166422. 27835699 10.1371/journal.pone.0166422 PMC5105946

[42] Wei J , Xu Z , Chen X , et al. Overexpression of GSDMC is a prognostic factor for predicting a poor outcome in lung adenocarcinoma. Mol Med Rep. 2020;21:360‐370. 31939622 10.3892/mmr.2019.10837 PMC6896373

[43] Hou J , Zhao R , Xia W , et al. PD‐L1‐mediated gasdermin C expression switches apoptosis to pyroptosis in cancer cells and facilitates tumour necrosis. Nat Cell Biol. 2020;22:1264‐1275. 32929201 10.1038/s41556-020-0575-z PMC7653546

[44] Park IH , Yang HN , Lee KJ , et al. Tumor‐derived IL‐18 induces PD‐1 expression on immunosuppressive NK cells in triple‐negative breast cancer. Oncotarget. 2017;8:32722‐32730. 28415798 10.18632/oncotarget.16281 PMC5464822

[45] Li K , Wei L , Huang Y , et al. Leptin promotes breast cancer cell migration and invasion via IL‐18 expression and secretion. Int J Oncol. 2016;48:2479‐2487. 27082857 10.3892/ijo.2016.3483

[46] Inoue N , Li W , Fujimoto Y , et al. High serum levels of interleukin‐18 are associated with worse outcomes in patients with breast cancer. Anticancer Res. 2019;39:5009‐5018. 31519608 10.21873/anticanres.13691

[47] He H , Yi L , Zhang B , et al. USP24‐GSDMB complex promotes bladder cancer proliferation via activation of the STAT3 pathway. Int J Biol Sci. 2021;17:2417‐2429. 34326684 10.7150/ijbs.54442 PMC8315027

[48] Tower H , Ruppert M , Britt K . The immune microenvironment of breast cancer progression. Cancers. 2019;11:1375. 10.3390/cancers11091375 PMC6769749 31527531

[49] Bareche Y , Buisseret L , Gruosso T , et al. Unraveling triple‐negative breast cancer tumor microenvironment heterogeneity: towards an optimized treatment approach. J Natl Cancer Inst. 2020;112:708‐719. 31665482 10.1093/jnci/djz208 PMC7357326

[50] Emens LA . Breast cancer immunobiology driving immunotherapy: vaccines and immune checkpoint blockade. Expert Rev Anticancer Ther. 2012;12:1597‐1611. 23253225 10.1586/era.12.147 PMC3587160

[51] Jain A , Song R , Wakeland EK , Pasare C . T cell‐intrinsic IL‐1R signaling licenses effector cytokine production by memory CD4 T cells. Nat Commun. 2018;9:3185. 30093707 10.1038/s41467-018-05489-7 PMC6085393

[52] Dinarello CA . Overview of the IL‐1 family in innate inflammation and acquired immunity. Immunol Rev. 2018;281:8‐27. 29247995 10.1111/imr.12621 PMC5756628

[53] Wang Q , Imamura R , Motani K , Kushiyama H , Nagata S , Suda T . Pyroptotic cells externalize eat‐me and release find‐me signals and are efficiently engulfed by macrophages. Int Immunol. 2013;25:363‐372. 23446850 10.1093/intimm/dxs161

