# Supplementary material for: Pyroptosis‐related molecular classification and immune microenvironment infiltration in breast cancer: A novel therapeutic target
Source: J Cell Mol Med. 2022 Mar 1;26(8):2259–72. doi: 10.1111/jcmm.17247 (PMC8995442; doi:10.1111/jcmm.17247)
Supplement: Supplementary file 1 — Supplementary Material [file JCMM-26-2259-s001.pdf]

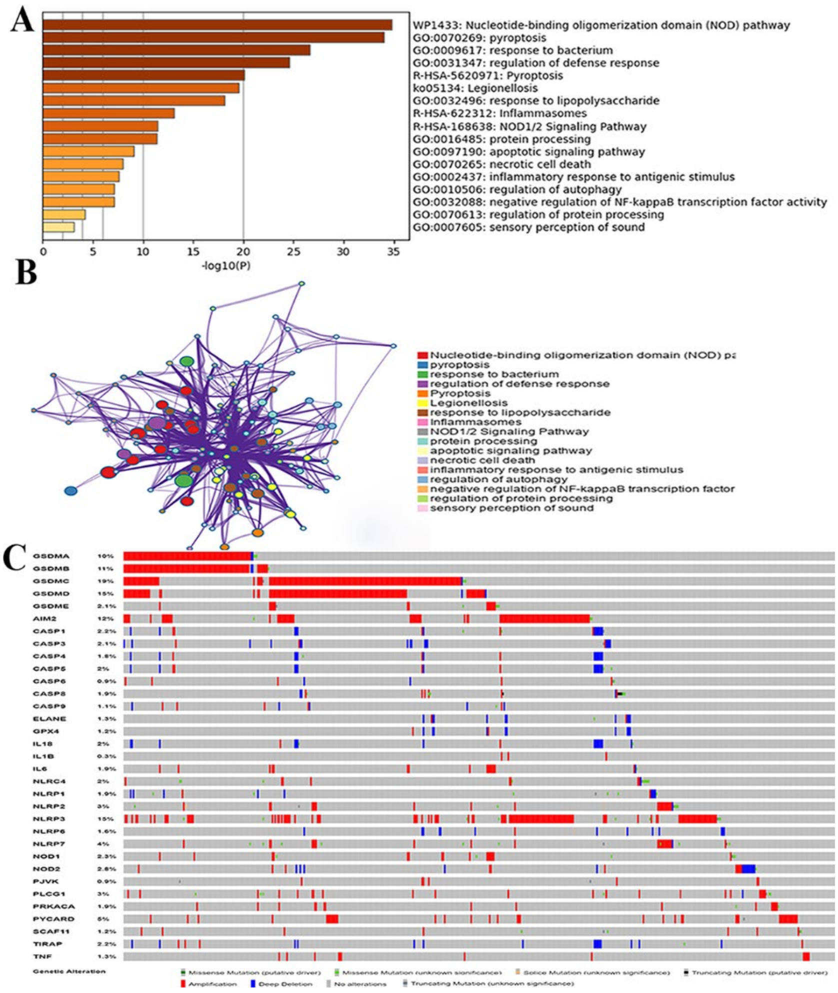

Supplementary Figure 1. Functional analysis and mutation information of 33 PRGs. (A) The enriched term in GO analysis, colored according to the p-value. (B) Network of enriched terms. (C) The landscape of genetic alteration profiles of PRGs in primary cohort. Different colors correspond to different mutation types and the annotations were at the bottom.

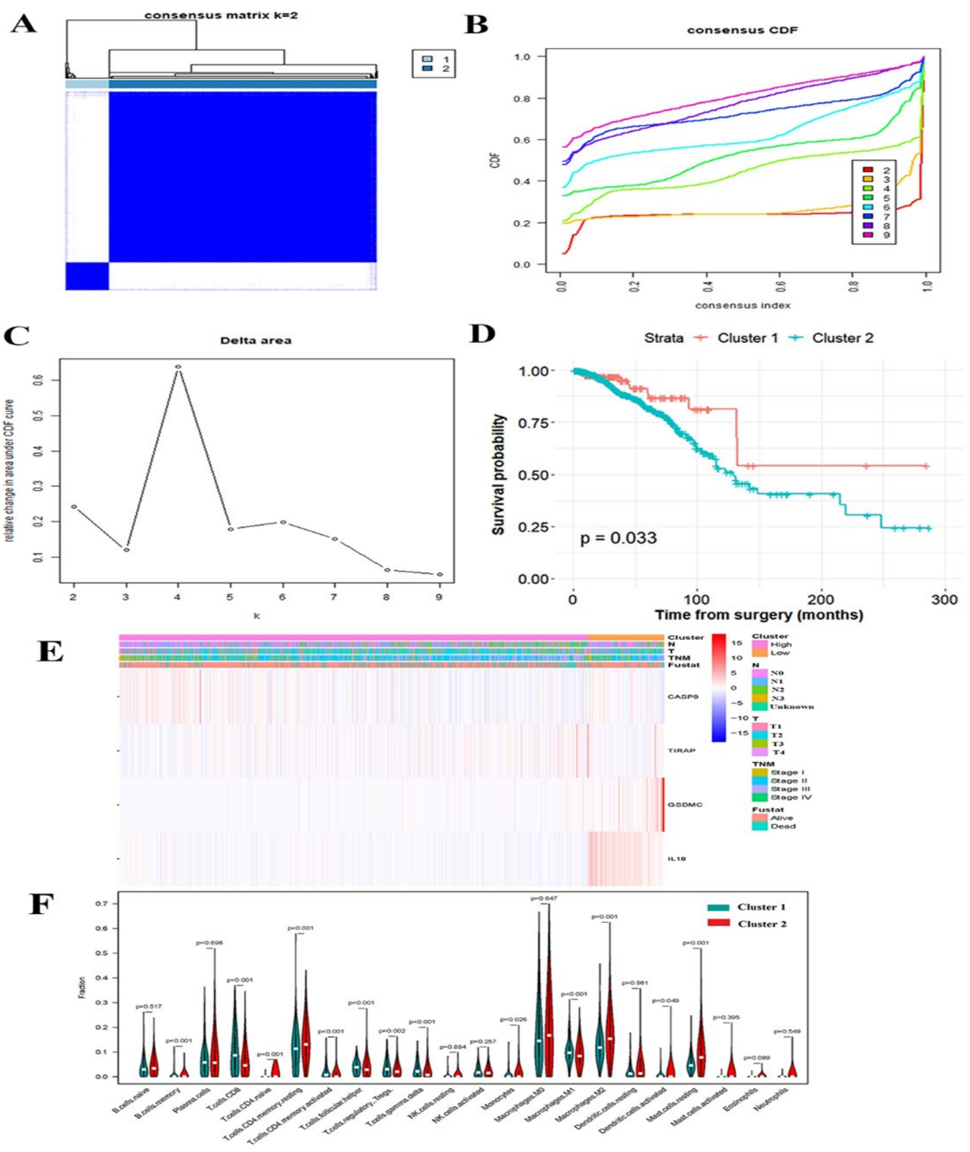

Supplementary Figure 2. Consensus clustering results and cluster characteristics of PRGs. (A) Consensus clustering matrix of PRGs (K=2). (B) Cumulative distribution functions in different iterations. (C) The proportion of ambiguous clustering matrix for k from 2 to 9. (D) Kaplan-Meier curves of OS between cluster 1 and cluster 2. (E) The clinicopathologic features of the two clusters are classified by the four PRGs. (F) The distribution differences of infiltration levels of 22 immune cell types in two clusters.
